# Beyond patient care: the impact of healthcare reform on job satisfaction in the Ethiopian public healthcare sector

**DOI:** 10.1186/s12960-017-0188-1

**Published:** 2017-02-03

**Authors:** Tsegahun Manyazewal, Mokgadi C. Matlakala

**Affiliations:** Department of Health Studies, College of Human Science, University of South Africa, PO Box 392, Pretoria, South Africa

**Keywords:** Healthcare reform, Job satisfaction, Health professionals, Ethiopia, Public healthcare sector, Moral satisfaction, Management style, Workload, Task

## Abstract

**Background:**

While healthcare reform has been a central attention for local governments, its impact on job satisfaction is poorly understood. This study aimed to determine the impact of healthcare reform on job satisfaction in the public healthcare sector in Ethiopia.

**Methods:**

The study was designed as a facility-based cross-sectional survey of healthcare professionals and carried out in all public hospitals in central Ethiopia which have been implementing healthcare reform (*n* = 5). All healthcare professionals in the hospitals who were involved in the reform from the inception (*n =* 476) were purposively sourced to complete a self-administered questionnaire adapted from a framework proposed for measuring job satisfaction of health professionals in sub-Saharan Africa. Kaiser-Meyer-Olkin and Bartlett’s tests were conducted to measure sampling adequacy and sphericity for factor analysis. Likert’s transformation formula was used to numerically analyse the satisfaction level of the respondents and to determine the cut-off value of satisfaction levels. Non-parametric and multiple logistic regression analysis were conducted to determine predictors of job satisfaction.

**Results:**

A total of 410 healthcare professionals completed the survey, representing an 88% response rate. The median and mean job satisfaction scores were 50 and 49, respectively, on a scale 1–100, which was equivalent to ‘Job dissatisfied’ on the Likert scale. Only 25% of respondents perceived job satisfaction due to implementation of the reform. Moral satisfaction (adjusted odds ratio (aOR), 177.65; 95% confidence interval (CI), 59.54–530.08), management style (aOR, 4.02; 95% CI, 1.49–10.83), workload (aOR, 2.42; 95% CI, 0.93–6.34), and task (aOR, 5.49; 95% CI, 2.31–13.07) were the most significant predictors. Job satisfaction results were significantly different among the study hospitals (*χ* ^2^ = 30.56, *p* < .001).

**Conclusions:**

The healthcare reform significantly and negatively influences public healthcare professionals’ job satisfaction and its overall impact on job satisfaction was poor, which would hinder the ‘Health Sector Transformation’ movement of Ethiopia. Healthcare reform efforts are contingent on job satisfaction of healthcare professionals, and such efforts should balance the demand and supply of both patients and providers for improved healthcare outcomes.

## Background

Healthcare professionals, as their patients, are clients of the healthcare system, in that their work requires the infrastructure, supervision, equipment, and physical setting to operate efficiently and cultivate a sustainable basic healthcare [1]. How well they respond to patients’ needs depends on various financial, professional, political, social, and personal factors [2]. In many countries, healthcare professionals are operating under serious policy, financial, organizational, and managerial constraints, with productivity, morale, and effectiveness suffering as a result. Principles for improving the values and skills of healthcare professionals were laudable but genuinely translating these into practice remained controversial. The recent World Health Organization global strategy on human resources for health: workforce 2030 has among its major principles to “uphold the personal, employment and professional rights of all healthcare workers, including safe and decent working environments and freedom from all kinds of discrimination, coercion, and violence” [3]. In essence, flourishing this into action thereby developing an emotionally strong, dedicated and job-satisfied healthcare professional that can go an extra distance to improve the quality of care will remain at the shoulder of each country.

There have been multiple healthcare reform initiatives in different settings that local governments were undertaking by virtue of their power to reform their healthcare environment for best outcomes on the delivery of care. Have these initiatives been following a two-tiered approach to satisfy the need of both patients and healthcare professionals, or rather affect job satisfaction levels of the professionals? Some scholars have complaints that healthcare reform efforts are sorely lacking better outcomes on job satisfaction [4, 5] while others reported for the reverse [6], but the extent and causes of the association between healthcare reform and job satisfaction are hidden by the absence of sufficient evidence. Healthcare professionals have been especially concerned about the nature of healthcare reform taking place in the healthcare sector as the reform could trigger major staff replacements, substitutions, and reduction which insists them to feel uncertainties about job security. This, in turn, would increase workload, compromise quality of care, and clouds the long-term visions of healthcare sectors.

In healthcare sectors that need to undergo healthcare reform, availability of resources and competency of workers are essential but not sufficient to ensure the desired worker performance. Rather, the effectiveness of the reform is critically dependent on worker job satisfaction and motivation of the Human Resource for Health (HRH) [7, 8]. Reengineering healthcare may lead to inadvertent voluntary resignations because of high dissatisfaction of health professionals with the process [9]. The surviving staff may experience low morale and motivation prompted by reactions such as insecurity, distrust, and anger which can also result in poorer patient outcomes [10]. For instance, healthcare providers reporting a greater number of hospital reengineering initiatives were also less satisfied with their jobs, less engaged in their work, more burned-out, and more likely to leave their job [11]. Job satisfaction brings to the healthcare professionals a pleasurable emotional state that often leads to a positive work attitude and improved performance and enables them to be altruistic, flexible, innovative, and loyal [12]. Healthcare leaders aiming to improve levels of job satisfaction should focus on workforce development and training efforts [5, 13]. Skilled healthcare professionals that practice under unattractive remuneration and in an operationally rigid conditions and living environment are at high attrition rates [14].

In Ethiopia, different Ethiopian regimes have been exploring acute healthcare reform strategies to satisfy health needs of the Ethiopian people. Succeeding the era of traditional medicine, the country has witnessed healthcare reform efforts including reform of modern health services, reforms of the national health policy and strategy, health reforms of the Provisional Government of the Socialist Ethiopia, health reform of the transitional government, and Business Process Reengineering (BPR) healthcare reform [15–21]. Each healthcare reform implemented at different times had its own contributions to the betterment of the health systems of the country. Starting in 2008, the Federal Democratic Republic of Ethiopia Ministry of Health (FMoH) has gone through a major country-wide healthcare reform initiative in the form of BPR. The purpose of the reform was to establish customer-focused institutions, rapidly scale up healthcare services, and enhance the quality of care in order to improve the health status of the Ethiopian people [18]. A system of tracking clients’ suggestions and complaints about services has been put in place to enable responsible departments of the FMoH to take appropriate and immediate actions. In line with this, the FMoH and agencies under the FMoH have identified seven core processes that need reengineering in order to effectively fulfil sectorial visions and missions. Each core process was organized with the major aim of bringing together similar activities in one place thereby decreasing lengthy service provisions and effectively utilizing resources. The reform has been progressively implemented through a series of training sessions for managers and technicians at all levels followed by changes in staff deployment, specific job assignments, and the recruitment of new staff. The FMoH, under its Improving Access and Quality of Health Service core process, has developed standards for specialized hospitals, primary hospitals, health centres, and health posts, and implemented in selected health facilities [21]. The Addis Ababa Health Bureau was among the administrative regions/city administrations in Ethiopia that have been customizing and implementing the reform that was developed by the FMoH to health facilities that it owned. When the bureau initiated this reform, though many of the city’s residents were public health facility users, the city’s health service coverage was 18% showing that the service was limited and seriously compromised [22]. Besides, as Addis Ababa is the capital city of Ethiopia, beyond the city’s permanent residents, patients across the country came to obtain better services. However, the healthcare services in the facilities were complicated and the way the facilities are structured was incapable to satisfy patients’ needs. In 2008, the Addis Ababa City Government Health Bureau (AAHB) conceptualized in detail previous and existing healthcare systems and implementation strategies using the BPR methodologies and identified major problems, causes, and effects. Among the major challenges identified were longer patient waiting time, multiple back and forth movement from one department to another, longer appointments, inadequate medical equipment and drugs, health care providers’ misbehaviour and incompetency, absence of accountability, demotivated staffs, incomplete services, and lack of information communication system between health facilities [22]. Based on these gaps, stretched objectives were synthesized and sub-processes that form the core process identified. Under its BPR implementation plan, the AAHB structured hospital services into three major case teams namely; Emergency, Outpatient, and Inpatient, where Outpatient and Inpatient case teams were further classified into eight and nine case teams, respectively. Each case team was expected to host technical, administrative and supporting staffs to enhance solidarity for a better healthcare provision. A general hospital and specialized teaching hospital were expected to host 416 and 450 clinical staffs, respectively [21, 22]. However, despite the fact that the Ethiopian government implemented the BPR healthcare reform across all public healthcare sectors in Ethiopia, the impact of the reform on job satisfaction of the health workforce was still poorly understood.

Thus, the study aimed to examine the impact of healthcare reform on job satisfaction in the public healthcare sector in central Ethiopia.

## Methods

### Setting and participants

The study was designed as a facility-based cross-sectional survey of health professionals and carried out in major and highly complex public hospitals in Addis Ababa, Ethiopia, between January to June 2015. Addis Ababa was selected among the 11 administrative divisions of Ethiopia considering its presence as the largest and city capital of Ethiopia and the political capital of Africa where the headquarters of the African Union, United Nations Economic Commission for Africa and numerous other continental and international organizations are based. The Addis Ababa Health Bureau administers six public hospitals which are anticipated to deliver advanced preventive and curative health services, from which all that have been implementing the BPR healthcare reform since its inception in 2008–2009 (*n =* 5) were purposively selected as study sites. The source population was all healthcare professionals that were working in the study sites at the time of data collection (*n =* 1681). Of this, all who were hired at least 1 year before the inception of the reform (*n =* 476, 28%) were drawn with a purposive sampling technique to select respondents who knew the performances of the hospitals before implementation of the reform and who could better analyse the changes that occurred due to the reform. The healthcare providers included medical doctors, laboratory professionals, nurses, health officers, pharmacists, dentists and sanitarians.

### Data instrument

The study instrument was a structured, self-administered questionnaire designed quantitatively in pursuance to a framework proposed to measure job satisfaction among health professionals in sub-Saharan Africa [23]. The framework involved eight domains of job satisfaction, namely, continuing education, salary and benefits, management style, tasks, work environment, workload, moral satisfaction, and job stability. Similarly, the central proportion of the study was the way the study got data, by asking healthcare professionals to assess the BPR healthcare reform in terms of job satisfaction. With this, each of the eight domains was represented by a specific item linked to a Likert-type scale, and the overall job satisfaction was a composite of the eight items (Table 1).

**Table 1:** Job satisfaction dimensions

Job satisfaction dimensions
Due to implementation of healthcare reform
*Continuing education*: Hospital management facilitates job-related training to staffs when necessary
*Task*: Staff have a clear job description that describes their routine duties in detail
*Salary*: Staff salary increases
*Workload*: Enough and competent health care workers and administrators are in place
*Management style*: Management involves the technical staff in decision making)
*Job stability*: Efficient and effective health care financing system has been established
*Work environment*: The way the hospital is structured is conducive to the daily workflow
*Moral satisfaction*: Staff are highly motivated to their work

Adapted from [23]

Independent of the eight domains, the respondents were asked in the questionnaire to rate their overall job satisfaction to separately assess their overall job satisfaction and use findings to further analyse job satisfaction predictors. The questionnaire also captured socio-demographic characteristics of the respondents, such as age, gender, duration of work as a health professional, duration of work as staff in the hospital, profession and level of education for conducting comparable analysis.

The study was granted Ethical Clearance Certificate from the Higher Degrees Committee of the Department of Health Studies, University of South Africa and the Addis Ababa and the Research and Technology Transfer Core process of the Addis Ababa City Administration Health Bureau. Written informed consents were developed for each respondent to read and sign before moving on to filling the questionnaires.

### Data analysis

Responses to the items in the eight domains were coded on a scale from 1 (strongly disagree) to 5 (strongly agree) and analysed numerically. The five scales were converted into a 0-to-100 scale by utilizing a Likert’s transformation formula [24].

$$ \mathrm{adjSS}=\kern0.5em 100\times \frac{\mathrm{stdSS}-1}{5-1} $$

where ‘adjSS’ and ‘stdSS’ are ‘adjusted satisfaction score’ and ‘standard satisfaction score’, respectively. With this scoring method, job satisfaction fell into five categories: ‘extremely dissatisfied’ (adjSS: 10–29), ‘dissatisfied’ (30–49), ‘generally satisfied or not’ (50–59), ‘satisfied’ (70–89), and ‘extremely satisfied’ (90–100).

On the other side, job satisfaction predictors were determined by computing overall job satisfaction score with job satisfaction domains. The item which was included to separately assess overall job satisfaction had original responses classified in Likert scale. Thus, the responses were dichotomized into ‘Job satisfied’ or ‘Job dissatisfied’ answers by taking the ‘Strongly agree’ and ‘Agree’ responses as a ‘Job satisfied’ value, while ‘Strongly disagree’, ‘Disagree’, and ‘Neutral’ responses as a ‘Job unsatisfied’ value. The output was used as the dependent variable to run logistic regression analysis to examine the correlation of job satisfaction with the domains of job satisfaction. Job satisfaction was taken as the dependent variable while the healthcare reform as the independent variable.

The instrument was pre-tested and Cronbach’s alpha [25] conducted to measure internal consistency. Kaiser-Meyer-Olkin and Bartlett’s tests [26] were conducted to ensure adequacy of sample size for factor analysis. Descriptive analyses were performed to provide background information of the data. Likert’s transformation formula was used to numerically analyse the data. Mann-Whitney *U* test [27], Kruskal-Wallis test [27], and multiple logistic regression analysis [28] were conducted to determine predictors of job satisfaction. Exploratory factor analysis [29] was conducted to determine the most significant factor of job satisfaction. Median and mean scores were used to measure the overall job satisfaction and the various job satisfaction sub-scales.

## Results

### General characteristics

Of the total eligible participants (*n =* 476), the questionnaire was distributed to those who consented (*n =* 465) and 88% (*n =* 410) were retrieved and those which were complete (*n =* 406) presented for analysis. The majority of the respondents (84.2%) had at least first degree and most of them (74.9%) were nurses followed by medical doctors (8.6%) and medical laboratory professionals (5.9%). A large number of participants (49.8%) had worked as a healthcare professional for 10 to 19 years, while a very small number of respondents (0.7%) had worked as a healthcare professional for 30 to 39 years. The general characteristics of the study respondents are presented in Table 2.

**Table 2:** Socio-demographic characteristics of respondents

Item	Count (*n =* 406)	%	Cumulative %
Gender			
Male	124	30.5	30.5
Female	282	69.5	100.0
Age (year)			
20–29	93	22.9	22.9
30–39	195	48.0	70.9
40–49	92	22.7	93.6
50–59	26	6.4	100.0
Duration of work as health professional (year)			
6–9	146	36.0	36.0
10–19	202	49.8	85.7
20–29	55	13.5	99.3
30–39	03	0.7	100.0
Duration of work as staff in this hospital (year)			
6–9	247	60.8	60.8
10–19	136	33.5	94.3
20–29	21	5.2	99.5
30–39	02	0.5	100.0
Profession			
Medical doctor	35	8.6	8.6
Laboratory	24	5.9	14.5
Pharmacy	16	3.9	18.5
Nurse	304	74.9	93.3
Health officer	14	3.4	96.8
X-ray technician	11	2.7	99.5
Sanitarian	2	0.5	100.0
Level of education			
Certificate	2	0.5	0.5
Diploma	37	9.1	9.6
Degree	342	84.2	93.8
MSc/MA or MPH	7	1.7	95.6
Medical doctor degree + speciality	18	4.4	100.0
Total	406	100	100

### Reliability and exploratory factor analysis

Kaiser-Meyer-Olkin test had a value of .795, which indicates that the sample size was statistically significant for factor analysis. The Cronbach’s alpha test had a value of .832, which satisfactorily ensured internal consistency of the data collection instrument. The exploratory factor analysis indicated that 75% of the variance has been explained by three variables among the eight job satisfaction domains.

### Job satisfaction scores

The median and mean job satisfaction score of all the respondents were 50 and 49, respectively, on a scale 1–100, which was equivalent to ‘Job dissatisfied’ on the Likert scale. From the five Likert categories, 133 (32.8%) were extremely dissatisfied, 169 (41.6%) generally satisfied or not, 91 (22.4%) satisfied, and 13 (3.2%) extremely satisfied. Only 25% of respondents were job satisfied as a result of the healthcare reform. Analysis of the Kruskal-Wallis test indicated that there is a significant difference in job satisfaction among the five study hospitals (*χ* ^2^ = 30.56, *p* < .001) (Table 3).

**Table 3:** Kruskal-Wallis test rank and test statistics of job satisfaction among study hospitals

Rank				Test statistics	
Hospital	Frequency	Mean rank	Sum of ranks	Number	406
1	72	228.44	16 447.68	Median	50.00
2	56	225.27	12 615.12	Chi square	30.557
3	99	227.87	22 559.13	*df*	4
4	71	156.08	11 081.68	Asymp. sig	0.000
5	108	184.42	19 917.36		
Total	406	203.50	82 620.97		

### Descriptive analysis

Of the total 406 healthcare professionals who responded to the questionnaire, 221 (54.4%) respondents claimed that there were no improvements in instituting continuing education programs in the hospitals. On the other hand, 188 (46.3%) respondents agreed that there were task flow systems in the hospitals that clearly describe in detail job descriptions of staff. While 256 (63.1%) of respondents argued that salary increment has not been made in line with the healthcare reform (Table 4), and 181 (44.6%) respondents agreed that there are improvements in staff workloads. Regarding the existing management style, 198 (48.8%) respondents argued that the management system of the hospitals is not participatory and only 96 (23.6%) respondents perceived job motivation due to the reform.

**Table 4:** Results based on dimensions of job satisfaction

Job satisfaction dimensions	Level of agreement		
	Agree f (%)	Neutral f (%)	Disagree f (%)
Continuing education	142 (35)	43 (10.6)	221 (54.4)
Task	188 (46.3)	96 (23.6)	122 (30)
Salary	93 (22.9)	57 (14)	256 (63.1)
Workload	181 (44.6)	98 (24.1)	127 (31.3)
Management style	153 (37.7)	55 (13.5)	198 (48.8)
Job stability	141 (34.7)	184 (45.3)	141 (34.7)
Work environment	161 (39.7)	110 (27.1)	135 (33.3)
Moral satisfaction	96 (23.6)	77 (19)	233 (57.4)
Total % score	36%	22%	42%

Due to the healthcare reform, the healthcare providers achieved an overall job satisfaction mean score of 2.87/5. Comparing mean scores of the eight job satisfaction dimensions, the highest mean scores were reached in workload (3.19) followed by task (3.18), while the lowest mean scores were observed in salary (2.35), followed by moral satisfaction (2.46).

**Fig. 1:**
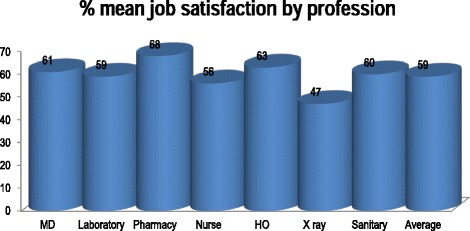
Cross-tabulation of job satisfaction by profession

Analysis of the Kruskal-Wallis test indicated that gender, age, duration of work as a health professional, duration of work within the hospitals, profession and level of education had no significant influence on job satisfaction (*p* = .099, .684, .761, .105, .144, and .309, respectively). However, comparing job satisfaction among professions with descriptive analyses, the highest mean scores of satisfaction were observed in pharmacy profession (3.38/5) followed by health officers (3.17/5), while the lowest mean scores of satisfaction were observed in X-ray professionals (2.34/5), followed by nurses (2.82/5). Figure 1 described cross-tabulation of percentage mean score of job satisfaction by profession.

### Predictors of job satisfaction

Table 5 presents eight independent variables of job satisfaction. In the bivariate logistic regression analysis, continuing education, task, salary workload, management style, job stability, work environment and moral satisfaction showed statistically significant association with job satisfaction. All the eight independent variables had significant associations with job satisfaction at a 5% level of significance. However, in the backward stepwise multivariate logistic regression analysis, only four variables have shown significant and independent association with job satisfaction, which were as follows: task (*p* < .001, adjusted odds ratio (aOR), 5.49; 95% confidence interval (CI), 2.31–13.07), workload (*p* < .072, aOR, 2.42; 95% CI, 0.93–6.34), management style (*p* < .006, aOR, 4.02; 95% CI, 1.49–10.83), and moral satisfaction (*p* < .001, aOR, 177.65; 95% CI, 59.54–530.08). While the remaining four predictor variables, namely, continuing education, salary, job stability and work environment had no association with job satisfaction in the multivariate logistic regression analysis (Table 5).

**Table 5:** Predictors of job satisfaction

		Job satisfaction					
Predictors		Frequency		*df*	Sig.	Crude OR (95% CI)	Adjusted OR (95% CI)
		Dissatisfied	Satisfied				
Continuing education	Poor	39	225	1	.906	1^R^ 5.934 (3.697, 9.524)	1.083 (0.291, 4.029)
	Good	72	70				
Task	Poor	21	197	1	.001^a^	1^R^ 8.615 (5.055, 14.684)	5.491 (2.307, 13.069)
	Good	90	98				
Salary	Poor	49	264	1	.099	1^R^ 10.776 (6.355, 18.271)	2.189 (0.864, 5.549)
	Good	62	31				
Workload	Poor	28	197	1	.072^b^	1^R^ 5.959 (3.643, 9.747)	2.422 (0.925, 6.342)
	Good	83	98				
Management style	Poor	40	213	1	.006^a^	1^R^ 4.611 (2.900, 7.331)	4.017 (1.490, 10.828)
	Good	71	82				
Job stability	Poor	42	223	1	.286	1^R^ 5.088 (3.191, 8.114)	1.769 (0.620, 5.049)
	Good	69	72				
Work environment	Poor	32	213	1	.798	1^R^ 6.413 (3.955, 10.397)	1.148 (0.400, 3.299)
	Good	79	82				
Moral satisfaction	Poor	21	289	1	.001^a^	1^R^ 206.429 (80.828, 527.2)	177.654 (59.539, 530.08)
	Good	90	06				

^a^Significant at 0.050; ^b^marginally significant at 0.050

## Discussion

The aim of this study was to examine the impact of healthcare reform on job satisfaction in the public health sector in Ethiopia. The results revealed that healthcare reform significantly and negatively influences public healthcare workforces’ job satisfaction. The overall job satisfaction of the workforce was low, which was consistent with previous studies that focused on public healthcare professionals in Ethiopia [30–33] and other countries in sub-Saharan African such as in Kenya [34], South Africa [35], Uganda [36, 37] and Ghana [38].

The study revealed that the majority of the job dissatisfactions were due to moral dissatisfaction, management style, workload, and task. These four dimensions are priority areas that the government and partner institutions should involve to ensure a job-satisfied staff across the public healthcare workforce in the country. The statistical analysis we conducted indicates no significant difference in job satisfaction among different gender, age, duration of work as a health professional, profession and level of education (*p* > .05). However, the descriptive analysis showed a higher sense of job dissatisfaction with X-ray professionals than the other healthcare professions, which could be due to physical working conditions of X-ray departments [39]. Pharmacy professionals were the most satisfied with their job, which concurs with the findings of another study held in Ethiopia [40]. Hence, a high level of effort is needed to improve job satisfaction of public healthcare staff, but the level of efforts needed among the different professions could be different. Healthcare professionals that perceived better job satisfaction due to implementation of the reform might be those that were transferred to a better position as a result of the reform thought there was no statistically significant evidence emerged from this study.

Staff moral dissatisfaction was recognized in the current study as the most salient problem and took the highest share towards job dissatisfaction; which was in line with previous studies conducted in Ethiopia [32, 41–43] and other East African countries [44]. According to the FMoH 2015 annual performance report, despite several gains in the HRH capital and leadership in Ethiopia, delay in the implementation of health workers motivation and retention packages and ineffective HRH management are challenging the system. Moral identities are dependent on the recognition of staff’s own needs for professional satisfaction and care [45]. In the presence of multiple HRH challenges, healthcare providers in resource-limited settings were shown enthusiasm to perform, which may serve as a motivator for them to improve their performance and that of the overall health sector [46]. Recognition of the public health workforce to reward their work and behaviours is a fundamental need for them to extend their determinations towards meeting the intended goals of the hospitals. The recognition shall be made in such a way that it is relevant to the actual needs of the workforce. Regularly monitoring staff service performance data to reward and attract best-performing providers who bring innovative ideas and procedures.

The study provides evidence that despite implementation of the healthcare reform, the management style remains delicate. Policies and methods used to manage HRH are at the core of any sustainable solution to healthcare system performance [15]. HRH management should take healthcare reform effort as a key factor for reducing job dissatisfaction of the health workforce [47, 48] and build their technical competency as they have the largest share in the success or failure of a reform [49–52]. The hospital management needs to regularly re‐evaluate the skills of the workforce to identify and rectify any skill deficits or training requirements to use the workforce’s full potential to achieve their tasks. The management should develop and implement a clearly defined career path for all staff as indicated in the Continuing Professional Development (CPD) guideline for health professionals in Ethiopia [53]. Just as a capable healthcare workforce is needed, so are capable HRH managers to guarantee effective implementation and oversight of policies, norms, and procedures [3]. The management should insist the staff keep updating their knowledge through continuing medical educations, workshops, and conferences. The management should also strengthen communication throughout the hospitals to develop a culture that encourages innovation, collaboration, and the free exchange of ideas, while foster a positive and welcoming environment that can nurture a culture of respect, inclusion, and equal opportunity.

The workload in the health workforce adversely affects patient safety [54], value and quality of care [55, 56], and personal life of the workforce [57–59]. This study revealed that the healthcare reform efforts did not reduce the workload of the workforce. This may result in delayed care, adverse events, medication errors, patient falls, nosocomial infections and patient death. The likelihood of these consequences in public hospitals could be reduced by assigning competent health workforce that matches with the patient load. It is likely to minimize diagnosis, care and treatment errors through the deployment of competent and appropriate numbers of the health workforce. The Ethiopian FMoH reports an increasing trend in health workforce density in the country, though it is still far behind the minimum threshold required to ensure high coverage with essential health interventions [60].

Though salary, as an independent variable, had a significant association with job satisfaction at a 5% level of significance, it did not in the backward stepwise multivariate logistic regression analysis. This could be due to the establishment of private wings in public hospitals in line with the reform to contribute in financing the hospitals as well as staff. The private wing is meant to improve the quality and timeliness of services, especially on weekends, to help reduce the turnover of skilled manpower through additional compensations, and to motivate staff members to provide more and better services for an additional fee for those who can afford to pay. Until 2013, there were 45 public hospitals which opened private wing services nationwide [61]. If the private wing is implemented effectively throughout public hospitals in Ethiopia, job dissatisfaction resulted from salary and benefits could decrease more. Besides, the recent public transportation the government has availed for public servants needs to be valued and supported.

This study had some limitations. The dependence of the study on healthcare respondents in hospitals, but not in lower level health facilities such as health centres, as primary sources of data could be a source of some limitations. The possible effect of this has been mitigated by inclusion of all public hospitals in the study area which have been implementing the reform from the inception, and as much of healthcare providers in the hospitals as possible. The respondents included were those who were hired before the inception of the reform to select respondents who knew the performances of the study sites before implementation of the reform and who could better analyse the changes that occurred due to the reform. Meanwhile, additional studies in other regions of the country are required to substantiate these findings.

## Conclusions

The results of this study provide new insight into the impact of healthcare reform on job satisfaction of public healthcare professionals. The healthcare reform significantly and negatively influences public healthcare professionals’ job satisfaction and its overall impact on job satisfaction was poor, which would hinder the ‘Health Sector Transformation’ movement of Ethiopia. The study revealed that the majority of job dissatisfactions were emerged from moral satisfaction, management style, workload, and task; thus, the four dimensions are priority areas that the government and partner institutions should involve to ensure a job-satisfied workforce across the public healthcare sector in the country. The conceptual terrain of this study marked that healthcare reform efforts are contingent on job satisfaction of healthcare professionals and such efforts should balance the demand and supply of both patients and providers for improved healthcare outcomes.

## Abbreviations

AAHB: Addis Ababa City Government Health Bureau
BPR: Business Process Reengineering
CPD: Continuing Professional Development
FMoH: Federal Democratic Republic of Ethiopia Ministry of Health
HRH: Human Resource for Health

## References

[1] Ding H, Sun X, Chang W-w, Zhang L, Xu X-p. A comparison of job satisfaction of community health workers before and after local comprehensive medical care reform: a typical field investigation in central China. PLoS ONE. 2013;8(9):e73438. 10.1371/journal.pone.0073438 PMC3772794 24058472

[2] Aluttis C, Bishaw T, Frank MW. The workforce for health in a globalized context-global shortages and international migration. Glob Health Action 2014;7. doi: 10.3402/gha.v7.23611. 10.3402/gha.v7.23611 PMC3926986 24560265

[3] World Health Organization. Global strategy on human resources for health: workforce 2030. Geneva: WHO; 2016.

[4] Taderera BH, Hendricks S, Pillay Y (2016). Health personnel retention strategies in a peri-urban community: an exploratory study on Epworth, Zimbabwe. Hum Resour Health.

[5] McIntosh T. Rolling-out Lean in the Saskatchewan Health Care System: Politics Derailing Policy. Health Reform Observer. 2016;4(1). http://dx.doi.org/10.13162/hro-ors.v4i1.2701.

[6] Fang P, Luo Z, Fang Z (2015). What is the job satisfaction and active participation of medical staff in public hospital reform: a study in Hubei province of China. Hum Resour Health.

[7] Khim K (2016). Are health workers motivated by income? Job motivation of Cambodian primary health workers implementing performance-based financing. Glob Health Action.

[8] Hung LM, Shi L, Wang H, Nie X, Meng Q (2013). Chinese primary care providers and motivating factors on performance. Fam Pract.

[9] Burke RJ, Ng EWS, Wolpin J (2011). Hospital restructuring and downsizing: effects on nursing staff well-being and perceived hospital functioning. Eur J Psychol.

[10] Duffield C, Kearin M, Johnston J, Leonard J (2007). The impact of hospital structure and restructuring on the nursing workforce. Aust J Adv Nurs.

[11] Buciuniene I, Blazeviciene A, Bliudziute E. Health care reform and job satisfaction of primary health care physicians in Lithuania. BMC Fam Pract. 2005;6(1):10. 10.1186/1471-2296-6-10 PMC555592 15748299

[12] Wicker D (2012). Job satisfaction: fact or fiction.

[13] Manyazewal T, Oosthuizen MJ, Matlakala MC (2016). Proposing evidence-based strategies to strengthen implementation of healthcare reform in resource-limited settings: a summative analysis. BMJ Open.

[14] Touré B, Avocksonma DA, Nyoni J, Ahmat A. Roadmap for scaling up human resources for health for improved health service delivery in the African Region 2012-2025. Afr Health Monitor 2013;18:20-26.

[15] Kitaw Y, Teka G, Meche H, Hailemariam D, Fantahun M (2012). The evolution of public health in Ethiopia.

[16] Mehari E, Gebeyehu K, Asfaw Z (2012). The manual of Ethiopian medical history.

[17] Tadesse L, Ardalan A (2014). Health sector initiatives for disaster risk management in Ethiopia: a narrative review. PLOS Curr Disasters.

[18] Federal Democratic Republic of Ethiopia Ministry of Health (FMoH) (2010). Health Sector Development Program IV, 2010/11–2014/14.

[19] Admasu K, Tamire A, Tsegaye S (2014). Envisioning the future of the health sector: an update. FMoH Q Health Bull.

[20] Federal Democratic Republic of Ethiopia Ministry of Health (FMoH) (2014). Ethiopia’s Fifth National Health Accounts 2010/2011.

[21] Federal Democratic Republic of Ethiopia Ministry of Health (FMoH) (2007). Standards for access and quality of health service.

[22] Adds Ababa City Administration Health Bureau (AAHB) (2008). Standard operation procedure for the delivery of medical services.

[23] Faye A, Fournier P, Diop I, Philibert A, Morestin F, Dumont A (2013). Developing a tool to measure satisfaction among health professionals in sub-Saharan Africa. Hum Resour Health.

[24] Liu JA, Wang Q, Lu ZX (2010). Job satisfaction and its modelling among township health centre employees: a quantitative study in poor rural China. BMC Health Serv Res.

[25] Mora M (2012). Research methodologies, innovations 1 and philosophies in software systems engineering and information systems.

[26] Denis DJ (2016). Applied univariate, bivariate, and multivariate statistics.

[27] Black K (2011). Business statistics: for contemporary decision making, 7^th^ edition.

[28] Hosmer DW, Lemeshow S, Sturdivant RX (2013). Applied logistic regression.

[29] Fabrigar LR, Wegener DT. Exploratory Factor Analysis: understanding statistics. New York: Oxford University Press; 2012.

[30] Geleto A, Baraki N, Atomsa GE, Dessie Y. Job satisfaction and associated factors among health care providers at public health institutions in Harari region, eastern Ethiopia: a cross-sectional study. BMC Res Notes. 2015;8:394. 10.1186/s13104-015-1368-5 PMC4553936 26323549

[31] Negussie N, Berehe C (2016). Factors affecting performance of public hospital nurses in Addis Ababa region, Ethiopia. J Egypt Public Health Assoc.

[32] Negussie N (2016). Job satisfaction of nurses in Jimma University Specialized Teaching Hospital, Ethiopia. J Egypt Public Health Assoc.

[33] Surur AS, Teni FS, Girmay G, Moges E, Tesfa M, Abraha M (2015). Assessment of the structural and process aspects of pharmaceutical care at a university hospital in Ethiopia. J Pharm Bioallied Sci.

[34] Ojakaa D, Olango S, Jarvis J (2014). Factors affecting motivation and retention of primary health care workers in three disparate regions in Kenya. Hum Resour Health.

[35] Blaauw D, Ditlopo P, Maseko F, Chirwa M, Mwisongo A, Bidwell P (2013). Comparing the job satisfaction and intention to leave of different categories of health workers in Tanzania, Malawi, and South Africa. Glob Health Action.

[36] Muliira RS, Ssendikadiwa VB (2016). Professional quality of life and associated factors among Ugandan midwives working in Mubende and Mityana rural districts. Matern Child Health J.

[37] Opollo JG, Gray J, Spies LA (2014). Work-related quality of life of Ugandan healthcare workers. Int Nurs Rev.

[38] Bonenberger M, Aikins M, Akweongo P, Wyss K (2014). The effects of health worker motivation and job satisfaction on turnover intention in Ghana: a cross-sectional study. Hum Resour Health.

[39] Magnavita N, Fileni A, Bergamaschi A (2009). Satisfaction at work among radiologists. La radiologia medica.

[40] Gebretekle GB, Fenta TG (2013). Assessment of pharmacists workforce in Ethiopia. Ethiop J Health Dev.

[41] Yami A, Hamza L, Hassen A, Jira C, Sudhakar M (2011). Job satisfaction and its determinants among health workers in Jimma University Specialized Hospital, southwest Ethiopia. Ethiop J Health Sci.

[42] Engeda EH, Birhanu AM, Alene KA (2014). Intent to stay in the nursing profession and associated factors among nurses working in Amhara Regional State Referral Hospitals, Ethiopia. BMC Nurs.

[43] Hotchkiss DR, Banteyerga H, Tharaney M (2015). Job satisfaction and motivation among public sector health workers: evidence from Ethiopia. Hum Resour Health.

[44] van der Doef M, Mbazzi FB, Verhoeven C (2012). Job conditions, job satisfaction, somatic complaints and burnout among East African nurses. J Clin Nurs.

[45] Peter E, Simmonds A, Liaschenko J. Nurses’ narratives of moral identity: making a difference and reciprocal holding. Nursing Ethics. 2016. ahead of print. 10.1177/0969733016648206 27220717

[46] Lutwama GW, Roos JH, Dolamo BL (2012). A descriptive study on health workforce performance after decentralisation of health services in Uganda. Hum Resour Health.

[47] Asegid A, Belachew T, Yimam E. Factors influencing job satisfaction and anticipated turnover among nurses in Sidama zone public health facilities, South Ethiopia. Nurs Res Pract. 2014;2014:909768. doi: 10.1155/2014/909768. 10.1155/2014/909768 PMC3953615 24707397

[48] Leggat SG, Bartram T, Stanton P (2011). High performance work systems: the gap between policy and practice in health care reform. J Health Organ Manag.

[49] Ramanigopal CS, Palaniappan G, Hemalatha N, Murugan T (2011). Business process reengineering and its applications. Int J Manag Res Rev.

[50] Mlay SV, Zlotnikova I, Watundu S (2013). A quantitative analysis of business process reengineering and organizational resistance: the case of Uganda. Afr J Inf Syst.

[51] JHabib MN, Wazir MI (2012). Role of education and training in the successful implementation of business process reengineering: a case of public sector of Khyber PakhtunKhwa (KPK). World J Soc Sci.

[52] Srikanth A (2012). Significance of BPR & ERP implementation in healthcare industry: an exploratory research. Int J Manag Strategy.

[53] Ethiopian Food, Medicine and Healthcare Administration and Control Authority (2013). Continuing Professional Development (CPD) Guideline for Health Professionals in Ethiopia.

[54] Chang LY, Yu HH (2016). The relationship between nursing workload, quality of care and nursing payment in intensive care units. Nurs Inf.

[55] Sennett C (2010). Healthcare reform: quality outcomes measurement and reporting. Am Health Drug Benefits.

[56] Matlakala MC, Botha AD (2016). Intensive care unit nurse managers’ views regarding nurse staffing in their units in South Africa. Intensive Crit Care Nurs.

[57] Nomura K, Yamazaki Y, Gruppen LD, Horie S, Takeuchi M, Illing J (2015). The difficulty of professional continuation among female doctors in Japan: a qualitative study of alumnae of 13 medical schools in Japan. BMJ Open.

[58] Sansom A, Calitri R, Carter M, Campbell J (2016). Understanding quit decisions in primary care: a qualitative study of older GPs. BMJ Open.

[59] Liu Q, Tian X, Tian J, Zhang X (2014). Evaluation of the effects of comprehensive reform on primary healthcare institutions in Anhui Province. BMC Health Serv Res.

[60] Federal Democratic Republic of Ethiopia Ministry of Health (FMoH) (2015). Health Sector Transformation Plan HSTP: 2015/16-2019/20.

[61] Elias N, Accorsi S (2013). Countdown to 2015: the performance of the health sector in the second year of HSDP IV (EFY 2004). FMoH Q Health Bull.

